# Development of idealized human aortic models for *in vitro* and *in silico* hemodynamic studies

**DOI:** 10.3389/fcvm.2024.1358601

**Published:** 2024-08-05

**Authors:** Hamid Mansouri, Muaz Kemerli, Robroy MacIver, Omid Amili

**Affiliations:** ^1^ Department of Mechanical, Industrial, and Manufacturing Engineering, University of Toledo, Toledo, OH, United States; ^2^ Department of Mechanical Engineering, Sakarya University, Sakarya, Turkey; ^3^ Children’s Heart Clinic, Children’s Hospitals and Clinics of Minnesota, Minneapolis, MN, United States

**Keywords:** aorta, idealized model, CAD model, *in vitro*, *in silico*, PIV, CFD

## Abstract

**Background:**

The aorta, a central component of the cardiovascular system, plays a pivotal role in ensuring blood circulation. Despite its importance, there is a notable lack of idealized models for experimental and computational studies.

**Objective:**

This study aims to develop computer-aided design (CAD) models for the idealized human aorta, intended for studying hemodynamics or solid mechanics in both *in vitro* and *in silico* settings.

**Methods:**

Various parameters were extracted from comprehensive literature sources to evaluate major anatomical characteristics of the aorta in healthy adults, including variations in aortic arch branches and corresponding dimensions. The idealized models were generated based on averages weighted by the cohort size of each study for several morphological parameters collected and compiled from image-based or cadaveric studies, as well as data from four recruited subjects. The models were used for hemodynamics assessment using particle image velocimetry (PIV) measurements and computational fluid dynamics (CFD) simulations.

**Results:**

Two CAD models for the idealized human aorta were developed, focusing on the healthy population. The CFD simulations, which align closely with the PIV measurements, capture the main global flow features and wall shear stress patterns observed in patient-specific cases, demonstrating the capabilities of the designed models.

**Conclusions:**

The collected statistical data on the aorta and the two idealized aorta models, covering prevalent arch variants known as Normal and Bovine types, are shown to be useful for examining the hemodynamics of the aorta. They also hold promise for applications in designing medical devices where anatomical statistics are needed.

## 1 Introduction

The aorta plays a vital role in the circulatory system as the largest arterial vessel, responsible for distributing oxygenated blood from the heart to the rest of the body (1). The hemodynamics (blood flow behavior) within the aorta may be strongly influenced by its geometric configuration (2). As such, for certain interventions or for the development of a personalized treatment plan, a 3D patient-specific reconstruction of the aorta (or part of the aorta) may be needed. However, to develop a generalizable understanding of the complex fundamental fluid mechanics in the aorta, aortic valve, and many other aspects of hemodynamics in both healthy and diseased cases, realistic but idealized models of the aorta or a related site are useful. Idealized models can facilitate the study of hemodynamics, offering insights applicable to broader populations, unlike patient-specific models. Additionally, idealized models are used for the development of computational and experimental methods and benchmarking data. This includes *in vitro* (experimental) studies such as particle image/tracking velocimetry (PIV/PTV), four-dimensional flow magnetic resonance imaging (4D-Flow MRI), and *in silico* (numerical) studies such as computational fluid dynamics (CFD), and more recently direct numerical simulation (DNS) of complex flow phenomena in the aorta or close sites. For example, in studying the local hemodynamics of the aortic valve, additional parameters such as ascending aorta diameter, sinus dimensions, annulus diameter, sinotubular junction height, coronary arteries diameters, and other dimensions of the aorta are required, highlighting the need for anatomical statistics. Examples of studies with idealized aortic geometries include (3–10).

Additionally, conducting comparative analyses in patient-specific geometries is often difficult due to the unique characteristics and distinct flow dynamics associated with each individual geometry (11). To address this common issue, one approach is to introduce an idealized model having the common features of the patient-specific anatomies, but yet generalizable to a large population. Such an idealized model can serve as a reference base case for both *in vitro* and *in silico* studies of the aorta and facilitating the design of medical devices. By employing such a model, it becomes possible to gain insight into the main characteristics of aortic blood flow and enhance our understanding of the dynamics of the circulatory system.

Furthermore, using an ideal model simplifies the complex manufacturing process of phantoms for physical experiments. The traditional fabrication method for *in vitro* hemodynamics involves manufacturing a phantom using clear silicon resin molding, e.g., (12). Another approach, made possible by recent advances in additive manufacturing methods, is 3D printing phantoms using a transparent material for optical imaging, e.g., (13), or using an opaque (or transparent) material for magnetic imaging, e.g., (14, 15). Additionally, a combination of 3D-printing and silicon modeling has been attempted recently (16). Overall, idealized models are easier to fabricate in both approaches due to their generally simpler geometry and superior surface smoothness. The smooth walls in generalized models potentially improve the imaging quality and minimize the light distortion and refraction in PIV/PTV experiments compared to segmented models. In numerical studies, due to the simplified model structure, grid generation becomes easier, resulting in improved mesh quality and solution convergence. Furthermore, idealized models can be easily modified digitally using various open-source or commercial CAD platforms since they are based on mathematical formulations as opposed to unstructured triangulated surfaces in patient-specific models that are based on biomedical imaging modalities.

We have examined the literature to determine the availability of any idealized models of the aorta. We noticed such models are very scarce, and among those available, the focus is on specific clinical conditions such as aortic dissection (17–19), ascending aortic aneurysm (20), and abdominal aortic aneurysm (21). Certain other studies had focused on specific sections of the aorta such as the abdominal aorta (22). Furthermore, the vast majority of these models tend to be an extremely simplified reconstruction of patient-specific cases, often adopting a straight tube, e.g., (6), or a basic U-bend tube configuration, e.g., (23), that neglects important anatomical factors such as curvatures and varying diameters of the thoracic aorta. For example, the model in the study by Vasava et al. (24) is based on a single patient data of Shahcheraghi et al. (25). Finally, to the best of the authors’ knowledge, none of the above studies shared an open-access CAD model or formulated a 3D geometry for use in aortic studies, whether experimentally or computationally, with the exception of the study by Liang et al. (20). In their study, they constructed a statistical shape model for the aortic curvature in patients with ascending aortic aneurysm, neglecting the branching vessels. In light of these limitations, the present study aims to address this gap by incorporating a comprehensive representation of reviewed anatomical features of healthy subjects into useful CAD models.

The anatomy of a healthy aorta has diverse applications, including the investigation of hemodynamics and thrombi transport within the aorta. For instance, it aids in the cardiogenic embolic stroke risk assessment, stroke location prediction, and understanding of stroke etiology and arterial embolism (26, 27). Additionally, they are utilized in the context of venous-arterial extracorporeal membrane oxygenation (VA-ECMO) for acute cardiogenic shock patients (28), and in benchmarking numerical studies in cardiovascular settings for boundary conditions (29), as well as exploring the effect of blood rheology modeling in aorta (30). Moreover, investigations focusing on the aorta from a solid mechanics perspective, which involve determining stress distribution in the aortic wall through finite element modeling (FEM) or utilizing deep learning (31, 32), as well as fluid–structure interaction (FSI) simulations (33, 34), also draw advantages from studies involving healthy populations. Importantly, there has been a growing focus on studying hemodynamics in the aorta of heart failure patients implanted with a left ventricular assist device (LVAD), most commonly a continuous-flow pump. The complex hemodynamics is of interest from several perspectives, including understanding altered hemodynamics, transport of small and large inertial thrombi, aortic insufficiency prevention, and optimizing the cannula graft angle and position (4, 35–39). The shape and dimensions of the aorta in such LVAD patients involve a healthy aorta without any abnormalities.

The main objective of this study is to develop idealized aortic models for the general population, intended for use in experimental or numerical investigations of the hemodynamics or solid mechanics of the aorta. The utilization of the models contributes to an enhanced comprehension of cardiovascular hemodynamics and holds the potential for improving the quality of life for patients in clinical settings. The idealized models presented in this study are developed by considering various geometrical parameters of the aorta as documented in the existing literature. These parameters are extracted from publications that utilized diverse imaging techniques such as computed tomography (CT), magnetic resonance imaging (MRI), cardiovascular magnetic resonance imaging (CMR), echocardiography, computed tomography angiography (CTA), quantitative coronary angiography (QCA), or are based on cadaveric studies. The dimensions collected from these sources encompass a broad range of population samples, spanning from 17 to 89 years old, with a specific focus on healthy adults. The study is structured to provide details on the search workflow, data compilation, and model development. It is followed by an experimental and computational assessment of the hemodynamics within the models, as well as discussion and conclusions.

## 2 Materials and methods

### 2.1 Study selection and data extraction

To identify relevant studies pertaining to aortic morphology, a critical search was conducted across prominent scientific databases, including PubMed, Web of Science, and Google Scholar. The search strategy aimed to encompass a comprehensive range of literature by utilizing appropriate keywords that included: “aortic arch morphology”, “thoracic aorta size”, “aortic arch geometry”, “arch vessel size”, “arch vessel distance/spacing”, “arch curvature”, “aortic root”, “coronary artery size”, and “arch vessel angle”. This extensive set of keywords ensured a thorough exploration of the literature landscape. The search was primarily focused on literature published from the year 2000 to 2023, providing an up-to-date perspective on aorta morphology studies.

**Figure 1:**
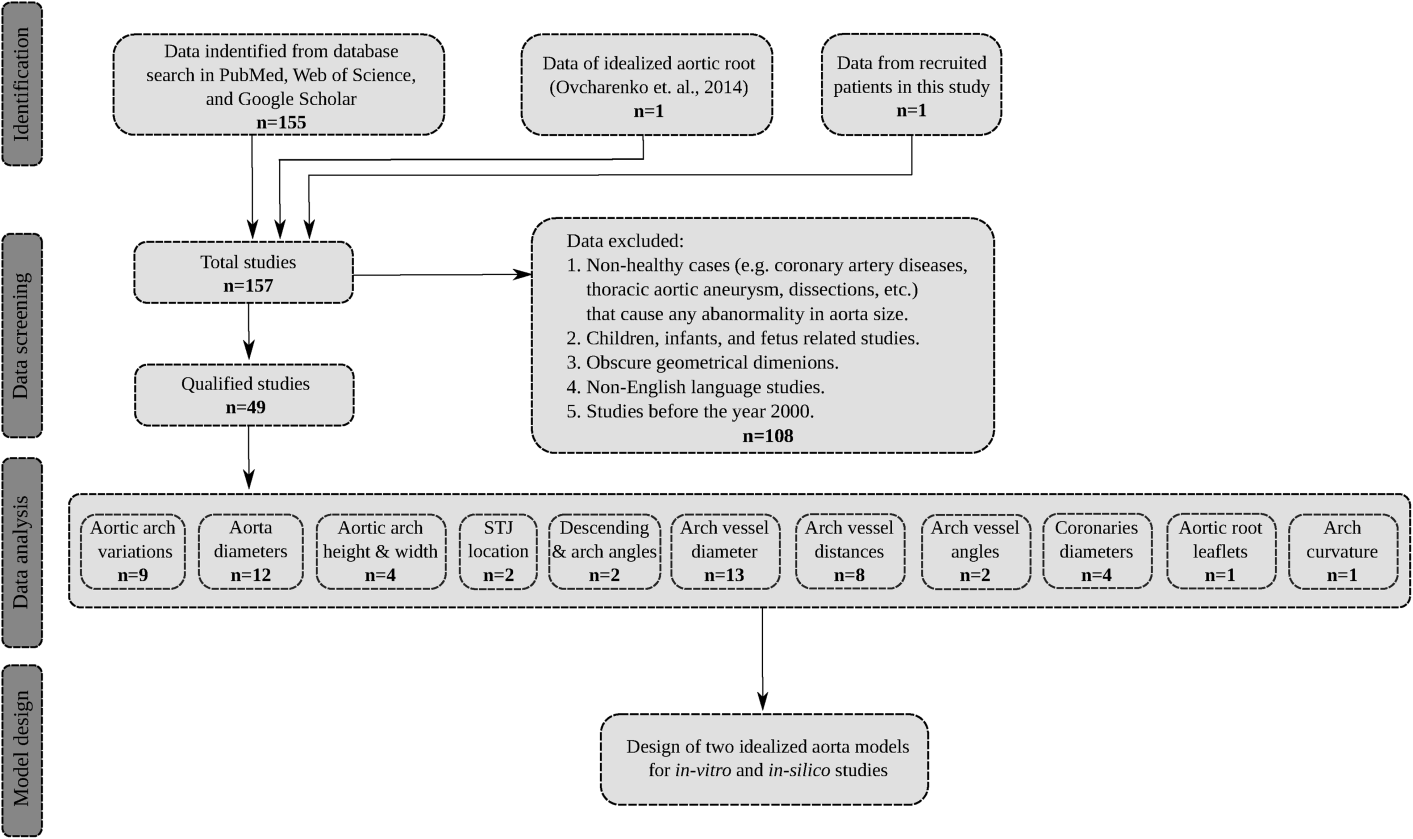
The flowchart of the search process, data extraction, and model development. The total number of qualified studies is n=49. Note that in the data analysis step, certain studies have reported more than one dimension of interest.

Initially, a total of 156 articles were identified through this screening. Subsequently, after reviewing the title, abstract, and the patient cohort, 108 articles were excluded from the study due to their focus on aortic size abnormalities in non-healthy patient populations such as coronary artery diseases, or thoracic ascending or abdominal aortic aneurysms and dissections. Additionally, articles that reported aortic dimensions in infants and children under the age of 16 were also excluded since such sizes can statistically differ from the adult population as suggested by Barmparas et al. (40) and Food et al. (41). Only studies that examined the aortic morphology in young, middle-aged, and elderly populations were considered. This included subjects spanning from 17 to 89 years old, and encompassed original research articles, review articles, and case reports, all of which were based on clinical imaging modalities or cadaveric investigations. Furthermore, non-English language studies were excluded from the final selection. The qualified studies were then categorized based on the various geometrical parameters of the thoracic aorta as reported in the following sections. By utilizing the average values of the identified parameters, weighted by the cohort size, two distinct aorta models were generated within a computer-aided design (CAD) environment. A flowchart showing the search, data collection and analysis is shown in Figure 1.

### 2.2 Data from recruited patients

In addition to the compiled data from the above search, anatomical dimensions of four patients recruited for a hemodynamics study by the authors were used to construct the aortic lumen models. High-resolution CT scan data of the patients were acquired after the institutional review board (IRB) approval, and were segmented using 3D Slicer® software, an open-source platform sponsored by the National Institutes of Health (NIH). Different dimensions were then extracted from the 3D models as reported in tables in Section 3 as well as a summary in Table 10.

### 2.3 Experimental study

To assess the hemodynamic performance of the idealized models developed in Section 4, we performed planar refractive-index-matched particle image velocimetry (PIV) measurements in a left ventricular assist device (LVAD) setting. Our choice has been inspired by the significance of hemodynamics within the aorta in LVAD patients as addressed in Section 1. We digitally grafted the device cannula, with a diameter of 16 mm, into the ascending aorta at a clinically common location and angle, and added a 0.6 mm wall thickness to the lumen, along with incorporating inflow and outflow fittings. The models were then 3D printed using stereolithography with Somos® WaterShed XC 11122 at 50 μm layers. Water was used as the working fluid, and the assessment of optical distortion showed sub-pixel accuracy attributed to the model’s thin wall thickness and smooth surface. To account for the viscosity difference between water and blood, we imposed inflow conditions corresponding to Reynolds numbers of approximately 1,757 and 3,163 (based on the inflow bulk velocity and the cannula diameter) as if the flow were blood stream at 5 LPM and 9 LPM, respectively. The steady-state inflow was distributed to different branches following the approach and rates described by Amili et al. (36), which utilized duplex ultrasound data.

The model was placed in a flow loop where its flow rate, pressure, and temperature were fully regulated and monitored. The flow was seeded with fluorescent green polyethylene beads and was illuminated using a light sheet generated by a continuous-wave laser at 405 nm. The light sheet with a thickness of approximately 2 mm was positioned at the symmetry plane of the inflow cannula, cutting through the model. This illumination was complemented by four DC LED lights at 415 nm. The seeded flow was then imaged using a Phantom T1340 high-speed camera in conjunction with a Zeiss lens at an f-number of 4.0, focused at the center plane of the light sheet. A sequence of 10,000 images was recorded at 200 Hz and 400 Hz for the low and high flow rates, respectively. For particle peak detection, a least-squares Gaussian fit (3×3 points) was employed. The interrogation window with the final pass size of 48×48 pixels with 75% overlap was used for the FFT-based cross-correlation.

### 2.4 Numerical study

A computational fluid dynamics (CFD) study of the hemodynamics was conducted within the idealized aorta models experimentally studied in Subsection 2.3, as well as the four patient-specific cases in Subsection 2.2. ANSYS Fluent steady-state k-ω based turbulent CFD solver was used with a tetrahedron mesh ranging from approximately 13 to 19 million elements, depending on the case. Inflation mesh layers were also defined at the lumen boundaries to better capture the near-wall gradients. The properties of blood were set to a dynamic viscosity of 0.004 Pa.s and a density of 1,060 kg/m^3^. An extension pipe with a length of 15 inlet diameter was added at the cannula inlet to ensure a fully developed inflow.

The inflow boundary condition was defined as the inlet velocity with a uniform bulk velocity corresponding to flow rates of 5 LPM and 9 LPM, matching the Reynolds numbers of Subsection 2.3. A uniform inflow turbulent intensity was applied at the inlet to match the bulk flow at the experimental conditions. The exit of the descending aorta was defined as a zero-pressure outlet, and the remaining vessels were described as mass flow rate outlets corresponding to the flow distribution of the experimental conditions. Second-order schemes were used for the spatial discretization, and the flow field was initialized with the hybrid initialization method in ANSYS Fluent solver. Simulations were performed at the Ohio Supercomputer Center (OSC), commonly at a node with 28 cores and 128 GB of memory.

## 3 Geometrical parameters of aorta

Dimensions of the aorta have long been utilized in the medical domain to facilitate the diagnosis and treatment of diverse cardiovascular conditions such as atherosclerosis or dilation (42, 43). However, such investigations have primarily focused on examining the general variability of geometric parameters across a broad population, rather than establishing a simplified and representative geometric model that accurately captures the complexity of the human aorta. In order to ensure broader applicability of proposed idealized models, the initial step involved identifying different types of aortic arches and presenting the statistical findings on the most prevalent arch variants. Subsequently, critical geometric parameters were precisely defined for the aorta and were subjected to a thorough analysis across a substantial population.

### 3.1 Variations of aortic arch branches

The anatomy of the aortic arch in humans exhibits diverse branching patterns that can vary significantly among individuals (44) and thus would lead to distinct hemodynamic characteristics. Understanding the variations in the type of aortic arch branches is essential for proposing a generalized ideal model to better represent the population. In the literature, three major types of aortic arches have been identified: Type I (also known as the Normal type), Type II (the Bovine type), and Type III (the Isolated type). The distribution of these three variants is presented in Table 1.

**Table 1:** Prevalence of different aortic arch branch types.

Authors	Cohort size (age [years])	Method	Normal type	Bovine type	Isolated type
			(Type I) [%]	(Type II) [%]	(Type III) [%]
Nelson and Sparks (45)	N=193 ^a^	Cadaver	94.3	1.03	3.1
Nayak et al. (46)	N=62 (45–79)	CT	91.4	4.8	1.6
Natsis et al. (47)	M=447, F=186 (19–79)	DSA	83	15	0.79
Jakanani and Adair (48)	N=643	CT	74	20	6
Celikyay et al. (44)	N=1,136	CT	74.7	21.1	2.9
Dumfarth et al. (49)	N=361	MSCTA	78.12	11.36	2.22
Huapaya et al. (50)	N=556	CT	66.5	24.6	6.3
Popieluszko et al. (51)	N=23,882 ^b^	Review	80.9	13.6	2.8
Recruited patients	N=4 (72.75±9.32)	CT	75	25	0
Weighted average			80.31	14.18	2.89

CT, computed tomography; DSA, digital subtraction angiography; MSCTA, multi slice CT spiral angiography; F, female; M, male.

^a^ Among the male population.

^b^ A systematic review of 51 different articles.

The Normal type is the most prevalent configuration at which the aortic arch gives rise to three major branches: the brachiocephalic artery (BA), left common carotid artery (LCA), and left subclavian artery (LSA). This type of aortic arch is present in approximately 80% of the general population. The Bovine type constitutes the second category where the BA and LCA arteries arise from a common trunk, while the LSA artery branches out from the aortic arch. This configuration comprises approximately 14% of the population. The Isolated type is characterized by the presence of four branches and is significantly less common with approximately 3% of the population. It is followed by several other uncommon variants of the aortic arch vessel configurations. The rare variants documented in the literature all combined cover almost 3% of the population. Therefore, our study primarily focuses on developing idealized models based on the first two variants.

### 3.2 Thoracic aorta dimensions

It is widely recognized that the dimensions of the thoracic aorta are influenced by various factors including gender, age, and body size (52). Despite variations, it is feasible to establish a general range with a reasonable standard deviation (53). This is owing to the fact that the increase in the thoracic aorta over 5 decades of aging is only 7 mm (54), and gender only puts on a 2 mm difference, corresponding to an approximate 7.5% increase in the aortic arch diameter, and this difference between genders decreases with age (55).

**Figure 2:**
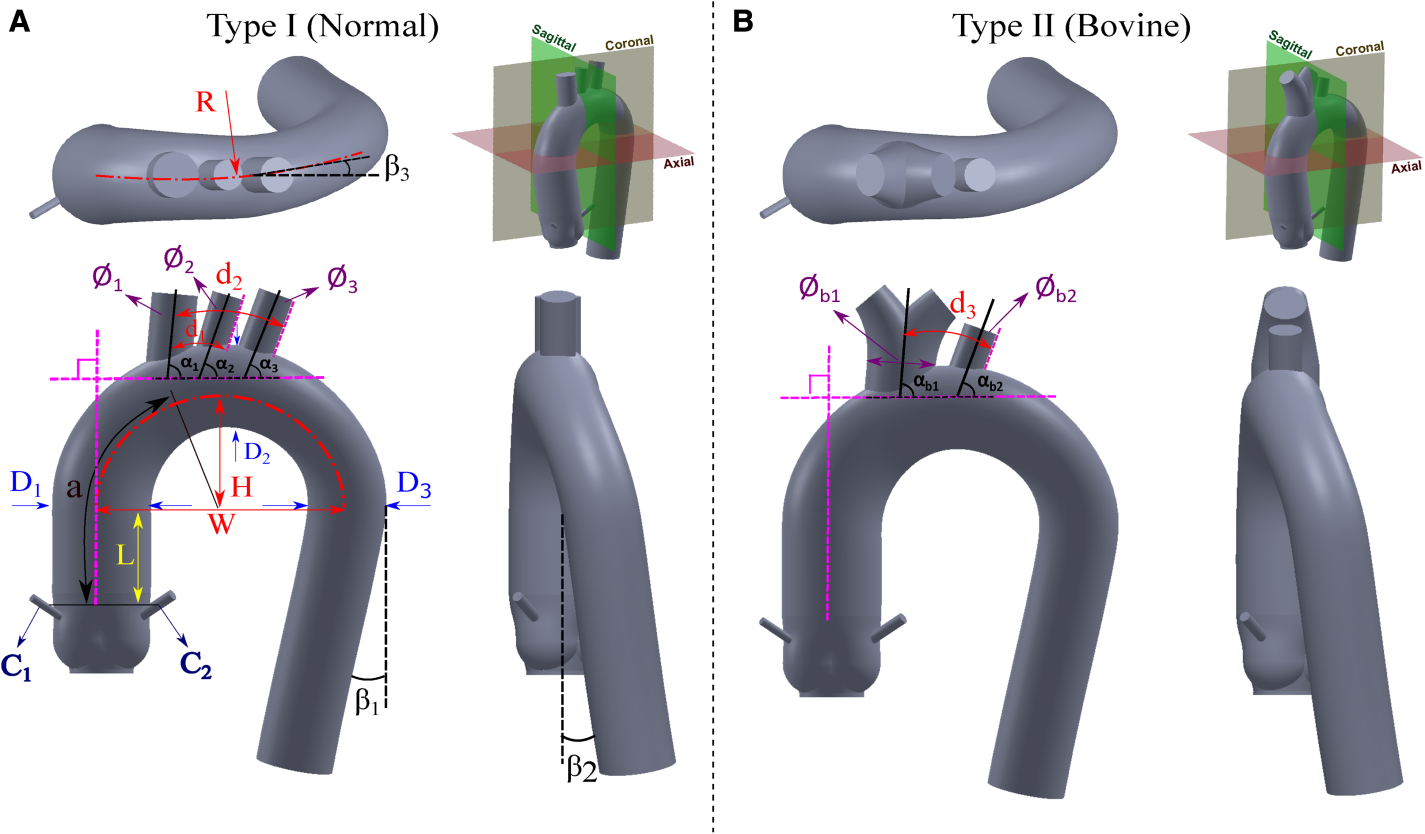
Proposed idealized models for the (**A**) Normal type (Type I), and (**B**) Bovine type (Type II).

We have reviewed the normal ranges of these dimensions and their variations in different populations as well as the measuring methods. By examining the variations of thoracic aortic dimensions towards clinical and research contexts, we have obtained the weighted average values of each section of the thoracic aorta presented in Table 2. We have focused on the five key dimensions which are the diameters of the aortic root, ascending aorta, aortic arch, proximal descending aorta, and mid-descending aorta. The diameters of the ascending aorta, aortic arch, and mid-descending aorta denoted as $D_{1}$, $D_{2}$, and $D_{3}$, respectively, are used for the developed idealized models as shown in Figure 2. These three dimensions suffice for a good representation of the aorta and are practical for CAD modeling of an idealized model since the aortic root diameter is very similar to the diameter of the ascending part, and the diameters of the mid-descending and proximal descending parts are also very similar to each other. The two parameters that are not considered in the idealized model development are reported in Table 2 for potential clinical use.

**Table 2:** Thoracic aorta dimensions.^a^

Authors	Cohort size	Method	Aortic root	Ascending part	Aortic arch	Proximal descending	Mid-descending
	(age [years])		[cm]	$D_{1}$ [cm]	$D_{2}$ [cm]	[cm]	$D_{3}$ [cm]
Hager et al. (53)	M=46, F=24 (17–89)	Helical CT	M=3.04, F=2.88	M=3.20, F=2.90	M=2.85, F=2.63	M=2.55, F=2.32	M=2.51, F=2.27
Wolak et al. (56)	N=4,039 (55±10.2)	CT	NA	3.3	NA	2.4	NA
Biaggi et al. (42)	M=815, F=984 (20–80)	TTE	M=3.40, F=3.10	M=3.20, F=3.00	NA	NA	NA
Evangelista et al. (57)	N=187	Echo	2.90	2.90	2.90	2.50	2.50
Redheuil et al. (54)	M=45, F=55 (20–70)	MRI	NA	M=3.10, F=3.00	NA	M=2.40, F=2.20	M=2.25, F=2.10
Craiem et al. (58)	N=51 (34–88)	CTA	NA	2.98	2.61	2.27	2.27
Davis et al. (59)	M=208, F=239 (19–70)	CMR	M=2.5, F=2.2	M=2.7, F=2.6	NA	M=2.1, F=1.9	NA
Vizzardi et al. (60)	M=495, F=507	Echo	M=2.63, F=2.40	M=2.59, F=3.39	M=2.59, F=2.44	NA	NA
Guo et al. (61)^b^	M=42, F=14 (58.2±17.9)	CTA	NA	3.20	2.73	2.54	2.47
Craiem et al. (62)	N=200 (58±9)	CT	NA	3	2.6	2.3	2.3
Zubair et al. (63)	N=116 (77.4±10)	CT	3.08	3.52	3.05	2.60	2.63
Recruited patients	N=4 (72.75±9.32)	CT	3.05	3.2	2.7	2.7	2.4
Weighted average			2.9	3.2	2.6	2.4	2.5

CMR, cardiac magnetic resonance; MRI, magnetic resonance imaging; Echo, echocardiography; TTE, transthoracic echocardiography; CT, computed tomography; CTA, computed tomography angiography; F, female; M, male.

^a^ Data has been reported based on the mean values and has the standard deviation range of 5-17% with respect to the mean.

^b^ Chinese population.

### 3.3 Height, width, and curvature of aortic arch

The morphology of the aortic arch typically exhibits a gradual curvature as it ascends from the aortic root and descends towards the descending aorta. The length and curvature of the aortic arch may vary among subjects and with age (54). To quantify these differences, it is common to measure the height (H) and width (W) of the aortic arch at a parasagittal plane. Additionally, the height-to-width ratio (H/W) serves as an important marker for identifying the elongation of the aorta. These parameters are applicable to both types of the aortic arch addressed in Subsection 3.1 and are illustrated in Figure 2. In addition to the height and width of the aortic arch, when viewing the aortic arch from a transverse (axial) plane, the arch slightly bends from the ascending side towards the descending side which is marked as R in Figure 2. Note that studies reporting this curvature dimension are very scarce; therefore we have used our recruited patients’ data as reported in Table 3.

**Table 3:** Height, width, and curvature of aortic arch.^a^

Authors	Cohort size (age [years])	Method	H [cm]	W [cm]	H/W	R [cm]
Redheuil et al. (54)	M=45, F=55 (20–70)	MRI	3.83	6.87	0.56	NA
Craiem et al. (62)	N=51 (34–88)	CTA	4.2	8.3	0.51	NA
Alhafez et al. (64)	N=120 (BAV), N=234 (TAV)	CT	4.1	8.2	0.5	NA
Recruited patients	N=4 (72.75±9.32)	CT	4.0	8.6	0.47	12.2
Weighted average			4.06	7.95	0.51	12.2

BAV, bicuspid aortic valve; TAV, tricuspid aortic valve; F, female; M, male.

^a^ Data has been reported based on the mean values and has the standard deviation range of 12-16% with respect to the mean.

### 3.4 Distance from sinotubular junction (STJ) to start of the aortic arch

This dimension starts from the end of the leaflets up to the point where the ascending aorta starts to bend and is shown by L in Figure 2. There is also an absolute lack of reporting of this dimension in the literature. Therefore, for the construction of both the Normal and Bovine idealized aortic models, we rely on our recruited patients’ data and the study by Vasava et al. (24) as summarized in Table 4.

**Table 4:** Distance from sinotubular junction to the beginning of the aortic arch.

Authors	Cohort size (age [years)]	Method	L [cm]
Vasava et al. (24)^a^	N=1 (young patient)	CTA	1.8
Recruited patients	N=4 (72.75±9.32)	CT	2.8
Weighted average			2.6

^a^ This study uses an idealized CAD model based on one patient for a CFD simulation, but the model is not available for public access.

### 3.5 Angles of descending aorta and aortic arch

The aortic arch has certain angles with respect to different axes that form the overall complex shape of the thoracic aorta. These angles are the two angles of rotation in descending aorta ($\beta_{1}$ and $\beta_{2}$) and one angle for aortic arch rotation in a transverse plane ($\beta_{3}$) as all shown in Figure 2. The angle $\beta_{1}$ of the descending aorta is measured in reference to a line tangent to the circumference of the mid-descending aorta. In addition, the descending aorta twists toward the posterior side with the angle of $\beta_{2}$. Moreover, when the thoracic arch traverses through the descending aorta, it deviates with an angle toward the posterior side denoted as $\beta_{3}$. Unfortunately, very limited data is available for these dimensions in the literature. To estimate the angle $\beta_{1}$, we rely on our recruited patients and the study by Vasava et al. (24). For the angles $\beta_{2}$ and $\beta_{3}$, we also extracted the data from our patients. All these three angles are shown in Table 5.

**Table 5:** Angles of descending aorta and aortic arch.

Authors	Cohort size (age [years])	Method	$\beta_{1}$ [^°^]	$\beta_{2}$ [^°^]	$\beta_{3}$ [^°^]
Vasava et al. (24)^a^	N=1 (young patient)	CTA	10	NA	NA
Recruited patients	N=4 (72.75±9.32)	CT	10	10	10
Weighted average			10	10	10

^a^ This study uses an idealized CAD model based on one patient for a CFD simulation, but the model is not available for public access.

### 3.6 Diameters of aortic arch vessels

The aortic arch gives rise to several major vessels that supply blood to the head, neck, and other organs through the brachiocephalic artery (BA), left common carotid artery (LCA), and left subclavian artery (LSA) of which their diameters are shown in Figure 2 as $\phi_{1}$, $\phi_{2}$, and $\phi_{3}$, respectively. In the case of the Bovine type, the BA and LCA branch out from a common trunk with a diameter of $\phi_{b1}$, while the diameter of LSA is denoted as $\phi_{b2}$. These three arteries, or in the case of the Bovine type aorta, the two branches, exhibit varying diameters among healthy individuals. Documenting the diameter of these branches is predominantly utilized in cardiovascular surgeries (65) such as the implementation of cerebral angiography catheter (66) as well as in procedures like radiological diagnostics and interventional radiology (67). Additionally, such diameter measurements are employed in monitoring the repair of the aortic arch following stent graft surgeries (68). The collected publications from the literature, along with the weighted averages with respect to the cohort size, are presented in Table 6. Of note, there is a limited number of studies focusing on the bovine type of aorta. It is also worth noting that in cases where the cross-sections of the aortic vessels exhibited an oval shape, the diameters are reported as the average of the larger and smaller diameters (65).

**Table 6:** Diameters of aortic arch vessels.^e^

Authors	Cohort size (age [years])	Method	Normal Type			Bovine Type	
			$\phi_{1}$ [cm]	$\phi_{2}$ [cm]	$\phi_{3}$ [cm]	$\phi_{b1}$ [cm]	$\phi_{b2}$ [cm]
Gupta and Sodhi (65)	N=77 (40–70)	Cadaver	0.87	0.61	0.67	1.65^a^	0.73^a^
Shin et al. (69)	N=25 (adult)	CT	1.83	0.98	1.06	NA	NA
Alsaif and Ramadan (70)	N=36 (adult)	CT	1.79	0.977	1.43	NA	NA
Vasava et al. (71)^f^	N=1 (adult)	CT	0.88	0.85	0.99	NA	NA
Rengier et al. (72)	N=20	CT	1.47	0.98	1.22	NA	NA
Finlay et al. (73)	N=45	CT	1.57	1.01	1.35	NA	NA
Carr et al. (26)	N=10 (≥65)	CT	1.35	0.75	1.03	NA	NA
Manole et al. (74)	M=24, F=9	CT	1.08	0.62	0.95	NA	NA
Osorio et al. (75)	N=1 (adult)	CT	1.24	0.74	0.74	NA	NA
Wilbring et al. (68)	N=118 (63±15)	CT	2.05	1.38	1.43	NA	NA
Zubair et al. (63)	N=116 (77.4±10)	CT	1.69	1.17	1.32	NA	NA
Tapia-Nañez et al. (76)	N=220 (52.7±17.6)	CT	1.28	0.86	1.08	2.21^b^	1.18^b^
Recruited patients	N=4 (72.75±9.32)	CT	1.25^c^	0.93^c^	1.35^c^	2.0^d^	1.58^d^
Weighted average			1.47	0.97	1.14	2.18	1.07

^a^ N=10.

^b^ N=30.

^c^ N=3.

^d^ N=1

^e^ Data has been reported based on the mean values and has the standard deviation range of 11-28% with respect to the mean.

^f^ This study uses an idealized CAD model based on one patient for a CFD simulation, but the model is not available for public access.

### 3.7 Distance between aortic arch vessels

The measurement of the spacing between the aortic arch vessels holds significant importance as a morphometric parameter within medical research. This parameter assumes crucial relevance in various aspects, including accurate diagnostics, treatment methodologies, and surgical interventions such as endovascular aortic stenting and catheterization (77). In order to provide a comprehensive understanding, a compilation of relevant parameters in the existing literature is presented in Table 7. Among the primary parameters under investigation, special attention is given to the distance between the BA and LCA arteries, denoted as $d_{1}$, as well as the distance between the LCA and LSA arteries, denoted as $d_{2}$. The distance between the first branch and the LSA artery in the Bovine type aorta is denoted as $d_{3}$. The distance from the sinotubular junction to the BA artery is also an important reference dimension that is denoted by a. All these parameters are illustrated in Figure 2.

**Table 7:** Distance between aortic arch vessels.^c^

Authors	Cohort size (age [years])	Method	a [cm]	Normal Type		Bovine Type
				$d_{1}$ [cm]	$d_{2}$ [cm]	$d_{3}$ [cm]
Gupta and Sodhi (65)	N=100 (68)	Cadaver	NA	1.54	3.02	2.51
Finlay et al. (73)	N=45 (68)	CT	7.77	1.83	4.74	NA
Wilbring et al. (68)	N=118 (63±15)	CT	7.7	1.69	3.31	NA
Liu et al. (78)	N=114 (53.3±14.4)	CT	7.82	NA	NA	NA
Zubair et al. (63)	N=116 (77.4±10)	CT	6.59	1.83	3.68	NA
Saade et al. (79)	N=75 (69±13.5)	CT	8.73	NA	NA	NA
Zerebiec et al. (66)	N=100 (62)	CT	NA	1.56	3.17	NA
Recruited patients	N=4 (72.75±9.32)	CT	7.5	1.73^a^	3.47^a^	4.00^b^
Weighted average			7.62	1.68	3.44	2.70

^a^ N=3.

^b^ N=1.

^c^ Data has been reported based on the mean values and has the standard deviation range of 23-28% with respect to the mean.

### 3.8 Angles of aortic arch vessels

The angle of the arch vessels is a significant morphometric parameter that plays a crucial role in hemodynamics and the potential blood clots transport to the head, especially in patients implanted with a left ventricular assist device (LVAD). Three angles are defined between the arch vessel centerline and the thoracic aorta. The angles of the BA, LCA, and LSA vessels with respect to the arch are denoted as $\alpha_{1}$, $\alpha_{2}$, and $\alpha_{3}$, respectively. For the Bovine type, the corresponding angles are presented as $\alpha_{b1}$ and $\alpha_{b2}$ for the first branch and LSA artery, respectively, as shown in Figure 2. The data is shown in Table 8.

**Table 8:** Angles of aortic arch vessels.^d^

Authors	Cohort size (age [years])	Method	$\alpha_{1}$ [^°^]	$\alpha_{2}$ [^°^]	$\alpha_{3}$ [^°^]	$\alpha_{b1}$ [^°^]	$\alpha_{b2}$ [^°^]
Demertzis et al. (80)	N=92 (69.4±9.9)	CT	84.79	73.9^a^	70.16	NA	NA
Recruited patients	N=4 (72.75±9.32)	CT	84^b^	57^b^	59^b^	80^c^	65^c^
Weighted average			84.77	73.31	69.81	80	65

^a^ N=83.

^b^ N=3.

^c^ N=1.

^d^ Data has been reported based on the mean values and has the standard deviation range of 12–22% with respect to the mean.

### 3.9 Aortic root

The aortic root serves as the anatomical connection between the left ventricle and the ascending aorta (81). The aortic valve, which normally has three leaflets (cusps), permits the passage of blood pumped from the contracting left ventricle. Its closure sustains the high pressure required in the systemic circulation. The shape and dimensions of the aortic root are adapted from Ovcharenko et al. (82) that collected data on subjects using CT and Echo image modalities, and generated an idealized aortic root model. We leveraged this study, which provided comprehensive data on parameters such as aortic root diameter, shape and depth of aortic sinuses, sinotubular junction diameter, and other relevant dimensions. This particular study is unique in the sense that its data is accessible online through the GrabCAD (83) platform, whereas the vast majority of reviewed studies do not provide any 3D segmented dataset or share a CAD file.

To ensure compatibility between the dimensions, the 3D model by Ovcharenko et al. (82) was scaled down by a factor of 0.9518 to fit into our compiled dimensions addressed in Section 3 and summarized in Table 10. This slight difference in the aortic root diameter naturally arises from different cohort sizes. The aforementioned study was also utilized as a point of reference for determining the location of the right coronary artery on the anterior coronary sinus and the left coronary artery on the left posterior aortic sinus.

### 3.10 Diameters of coronary arteries

The coronary artery dimension plays a significant role in diagnosing and treating various conditions such as coronary artery disease (84, 85). In surgical procedures such as percutaneous coronary intervention (PCI) and coronary artery bypass graft (CABG) surgery, the size of the coronary arteries holds particular importance (86). Typically, this size is considered independent of factors such as age and body size, except for gender (85, 87). Yet, certain studies have found no significant difference in coronary artery size based on gender (86). In both the Normal type and Bovine type aortas, the diameter of the coronary arteries is denoted as $C_{1}$ and $C_{2}$ for the right and left coronaries, respectively, as illustrated in Figure 2, and reported in Table 9.

**Table 9:** Diameters of coronary arteries.^a^

Authors	Cohort size (age [years])	Method	$C_{1}$ [mm]	$C_{2}$ [mm]
Cavalcanti et al. (88)	N=51	Cadaver	2.9	3.75
Mehrotra et al. (84)^b^	N=321 (49.4±11.22)	QCA	3.1	4.28
Raut et al. (86)	N=229 (51.7±9.35)	QCA	1.83	2.34
Recruited patients	N=4 (72.75±9.32)	CT	2	2
Weighted average			2.6	3.5

QCA, quantitative coronary angiography.

^a^ Data has been reported based on the mean values and has the standard deviation range of 20-27% with respect to the mean.

^b^ Indian population.

### 3.11 Aortic valve

The aortic valve, typically a trileaflet valve, has not been included in the model design because it is a dynamic component of the aorta-heart. The shape and opening configuration of its leaflets vary within the cardiac cycle. The operation of the valve leaflets may also vary depending on the condition or disease being studied. Additionally, valves can be native, transcatheter, or mechanical, with either two or three cusps. Given these variations, researchers can digitally integrate their choice of valves into the proposed models for *in vitro* and *in silico* studies. Several notable studies on the aortic valves, including those involving mechanical, bioprosthetic, or idealized models, are available (3, 6, 89–95).

## 4 Proposed idealized aorta models

Based on the literature survey reported here, we compiled and summarized the weighted averages of all relevant dimensions of the aorta in Table 10. We then used this table to design two idealized models, following the same naming convention in the literature, for the Normal type (Type I) and the Bovine type (Type II) of the aorta as shown in Figure 2. For each model, three views are used to clearly show all the dimensions as well as the fourth view positioned with respect to the standard anatomical planes. The Bovine type shares the same statistical dimensions as the Normal type with the exception of the arch vessels and their corresponding diameters and angles.

**Table 10:** A summary of the aorta dimensions derived from four recruited patients (Patients 1–4), and a summary of the weighted averages of aorta dimensions used for the two proposed idealized aorta models (Type I and Type II). Dimensions are in centimeters and angles in degrees. The cumulative number of subjects used for the weighted averages in the idealized models is denoted as N.

Models	$D_{1}$	$D_{2}$	$D_{3}$	$C_{1}$	$C_{2}$	$d_{1}$	$d_{2}$	$d_{3}$	$\Phi_{1}$	$\Phi_{2}$	$\Phi_{3}$	$\Phi_{b1}$	$\Phi_{b2}$	W	H	R	L	a	$\alpha_{1}$	$\alpha_{2}$	$\alpha_{3}$	$\alpha_{b1}$	$\alpha_{b2}$	$\beta_{1}$	$\beta_{2}$	$\beta_{3}$
Patient 1^a^	3.2	2.6	2.7	0.2	0.2	NA	NA	4	NA	NA	NA	2	1.58	9.2	4	9.2	2.4	8.5	NA	NA	NA	80	65	10	0	0
Patient 2^b^	3.3	2.75	2.35	0.2	0.2	1.8	3.54	NA	1.35	1	1.25	NA	NA	9.2	4	13.1	3.2	7.5	82	60	60	NA	NA	0	0	0
Patient 3^b^	3.15	2.8	2.25	0.2	0.2	1.7	3.4	NA	1.2	0.95	1.35	NA	NA	8	4	13.2	2.8	7	85	55	55	NA	NA	15	20	20
Patient 4^b^	3.15	2.7	2.25	0.2	0.2	1.7	3.4	NA	1.2	0.85	1.45	NA	NA	8	4	13.3	2.8	7	85	55	55	NA	NA	15	20	20
Average	3.2	2.7	2.4	0.2	0.2	1.73	3.47	4	1.25	0.93	1.35	2	1.58	8.6	4	12.2	2.8	7.5	84	57	59	80	65	10	10	10
Type I	3.17	2.65	2.5	0.26	0.35	1.68	3.44	NA	1.47	0.97	1.14	NA	NA	7.95	4.06	12.2	2.6	7.62	84.8	73.3	69.8	NA	NA	10	10	10
Type II	3.17	2.65	2.5	0.26	0.35	NA	NA	2.7	NA	NA	NA	2.18	1.07	7.95	4.06	12.2	2.6	7.62	NA	NA	NA	80	65	10	10	10
N	8,071	2,133	728	605	605	483	483	104	706	706	706	301	301	509	509	4	5	472	96	96	96	1	1	5	4	4

^a^ Classified as Type II.

^b^ Classified as Type I.

For the purpose of CAD modeling, SolidWorks® 2022 was utilized. First, we sketched circular profiles of the aortic models in multiple cross-sections. Then, the “*loft*” feature was leveraged to create a transition between the cross-section profiles while ensuring a smooth overall shape. The sketch was started from the cross-section of the STJ location and ended with the termination circle of the descending aorta. Additional intermediate profiles (circles) were necessary to refine the final shape of the models. Two guide curves that connected the profiles were drawn using the “*spline*” feature. Due to the arch curvature with a particular radius of curvature (R), the “*3D sketch*” feature was activated before using the “*spline*” drawing tool to establish a proper out-of-plane curvature. Then, the tangency directions at spline points were adjusted based on the models’ design. The arch vessels were then added to the model following the corresponding dimensions. Finally, the aortic sinuses along with the coronary arteries were added.

For each idealized aorta, we provide the CAD model for the lumen as shown in Figure 2, as well as a shell version with a thickness of 1.0 mm. The lumen model can be used in numerical simulations or to mold a phantom for experimental studies. The editable shell model with a finite wall thickness is suitable for the purpose of fabrication via additive manufacturing. All models are shared online in both STereoLithography (STL) and SolidWorks part (SLDPRT) file formats.

## 5 Hemodynamics analysis

**Figure 3:**
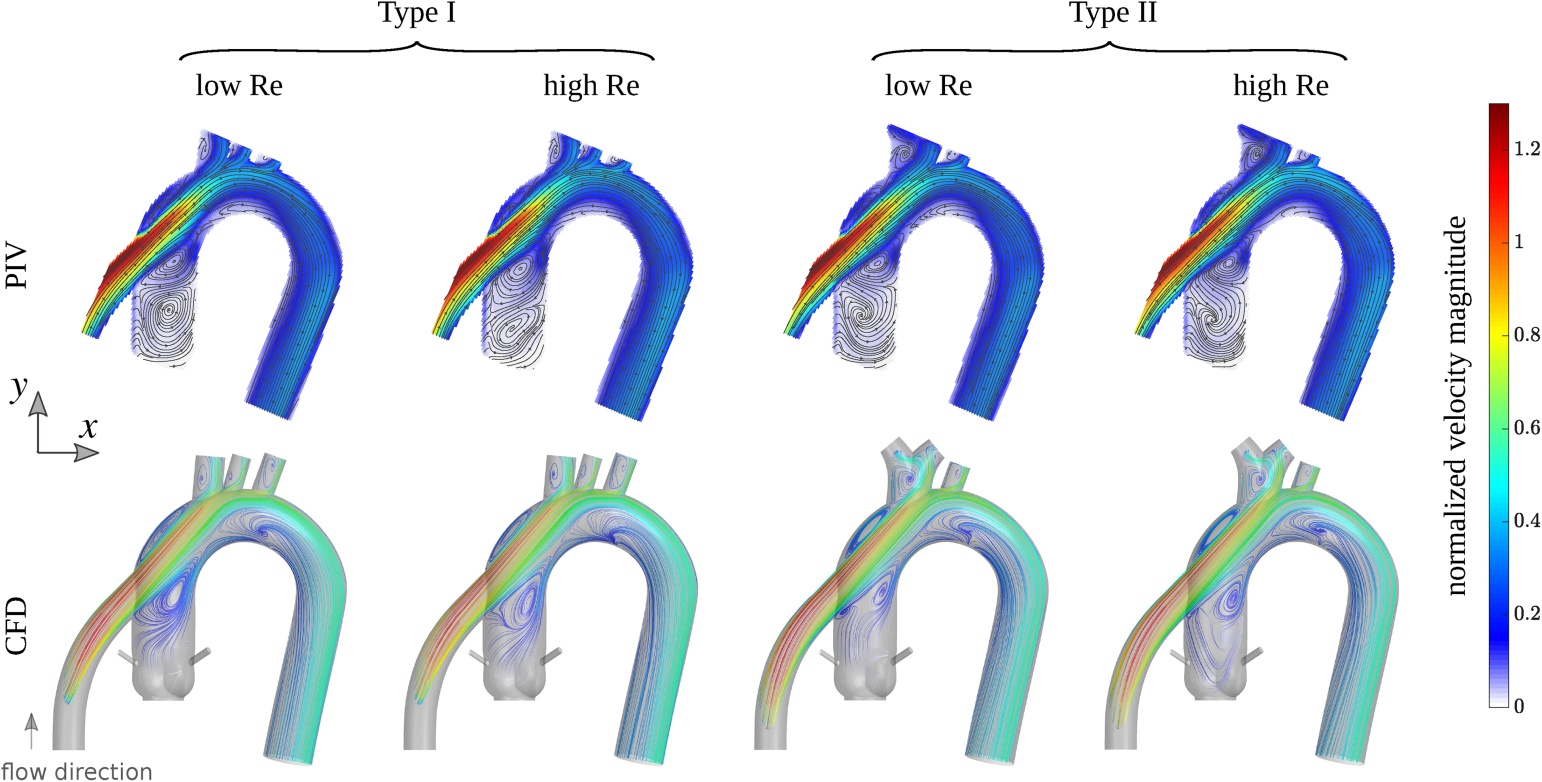
First row: streamlines overlaid on the velocity magnitude field measured by PIV for Type I and Type II idealized models grafted with the outflow of a left ventricular assist device (LVAD). Second row: streamlines from CFD colored by the velocity magnitude computed in a plane comparable to experiments. The colorbar shows the velocity magnitude normalized by the inflow bulk velocity of each case. The Reynolds number (based on the inflow bulk velocity and the cannula diameter) is approximately 1,757 and 3,163 for the low and high flow rates cases, respectively.

To assess the performance of our developed idealized models, we conducted high-resolution planar PIV experiments and high-resolution CFD simulations for the two models in a left ventricular assist device (LVAD) setting, as detailed in Subsections 2.3 and 2.4. We used two Reynolds numbers for both idealized models in both CFD and PIV settings, and one Reynolds number for the four patient-specific CFD cases, totaling 12 studies combined. The CFD results are first verified against PIV experiments, as shown in Figure 3, which depicts streamlines colored by the local velocity magnitude in the CFD cases, and streamlines overlaid on the measured velocity magnitude field in the PIV cases. The velocity magnitude was normalized by the inflow bulk velocity of each case. The incoming flow in the cannula accelerates towards the superior direction as it approaches the graft location due to its strong curvature, which is consistent in all cases. The jetting flow entering the aorta induces several recirculation zones, which are observed in both types of models. At the higher Reynolds number, these zones slightly change in size or intensity, but they remain remarkably persistent. While certain minor differences in the flow topology are noticed between CFD and PIV, it is important to note that the modalities are inherently different. Streamlines in PIV are based on 2D measurements (a 2D projection of the 3D velocity field), while CFD computations are fully 3D, and streamlines are computed on a plane comparable to that of PIV. For a detailed quantitative comparison, the probability density function (PDF) of the normalized velocity magnitude in idealized models obtained by CFD and PIV is shown in Supplementary Figure S1. Additionally, the median of the quantities along with their 25% and 75% percentiles is shown in the same figure. Notably, for a fair comparison, the CFD data was extracted from a comparable plane to the PIV plane, as shown in Figure 3.

**Figure 4:**
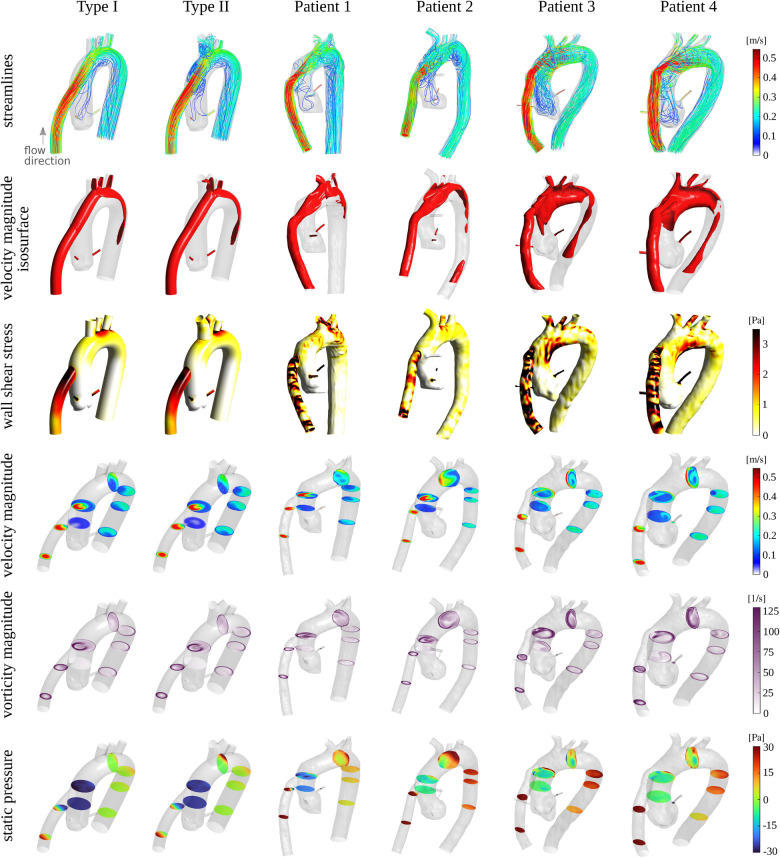
First row: streamlines colored by the local velocity magnitude. Second row: isosurface of the velocity magnitude at a threshold level of approximately 0.2 m/s corresponding to 50% of the bulk velocity at the inflow. Third row: wall shear stress map. Fourth, fifth, and sixth rows: cross-sections at different locations colored by the velocity magnitude, vorticity magnitude, and static pressure, respectively. The Reynolds number (based on the inflow bulk velocity and the cannula diameter) for all cases is approximately 1,757.

After establishing a close agreement between simulations and experiments, we compared the flow patterns in the idealized models with those in the four subjects in Subsection 2.2. Figure 4 illustrates the flow structure within the models from different perspectives. In the first row, streamlines colored by the local velocity magnitude depict the overall complex flow pattern, which is highly vortical near the aortic root. The second row illustrates the high-momentum flow structure within the cannula and the aorta using the isosurface of the velocity magnitude. We used 50% of the inflow bulk velocity as the threshold level for each isosurface. In the third row, the local wall shear stress map shows elevated values near the arch vessels, at the coronaries, and at the cannula. In the maps for the last two patients (3 and 4), an increased level of wall shear stress is also noticeable at the inferior region of the aortic arch. This is due to the different cannula angles compared to the other two patients (1 and 2) and the two idealized models. The cannula in the last two patients directs the jetting flow toward the posterior-inferior direction, where it impinges on the wall, causing an elevated wall shear stress region. To better illustrate the flow behavior, several cross-sections were extracted at different locations, showing velocity magnitude, vorticity magnitude, and static pressure in the fourth, fifth, and sixth rows, respectively. While certain differences are observed between the idealized models and the subject-specific models (particularly for patients 3 and 4), the global flow patterns and the range of quantities are consistent. The differences in fine flow features, especially in wall shear stress, which may be sensitive to the anatomical features, are expected. Notably, the idealized models show a smaller pressure drop between the cannula inlet and the descending aorta outlet. This is perhaps due to their simpler geometry as well as greater surface smoothness. For a detailed quantitative comparison, Supplementary Figure S2 presents probability density functions and percentile plots for velocity magnitude, vorticity magnitude, wall shear stress, and static pressure for all CFD cases at the 5 LPM flow rate setting.

## 6 Discussion

This study highlights the scarcity of idealized models for the aorta. Among the few available, the models are typically designed for specific clinical conditions such as aortic dissection, ascending aortic aneurysm, or abdominal aortic aneurysm (18–22), and are often overly simplified reconstructions based on a single patient case. To address this research gap, we conducted a critical literature review focusing on the morphometric parameters of the healthy human aorta. The surveyed data, along with the data from four recruited subjects were compiled to present a comprehensive summary of relevant dimensions and angles. Our study illustrates the population’s heterogeneity, showcasing diverse aortic types and sizes, while also highlighting the lack of data on specific dimensions such as the descending aorta angle with respect to anatomical planes and the branching angle of vessels from the aortic arch. To ensure a more realistic representation of dimensions, the average of each parameter was weighted by the study cohort size. While aortic size measurements, including those from the four recruited subjects, are derived from various imaging modalities such as CT and MRI, our review of the existing studies in Section 3 affirms that there is no significant difference between these measurements across imaging methods, which is consistent with the multi-modality assessment of thoracic aortic dimensions by Frazao et al. (96). They reported a great level of agreement in thoracic aortic measurements between CT and MRI. However, they also found that TTE significantly underestimates the maximum aortic root diameter compared to CT and MRI. In the present work, very few studies based on echo modalities were found and used in our workflow.

Subsequently, we utilized these dimensions to develop 3D CAD models of the aorta, incorporating both the Normal (Type I) and Bovine (Type II) aortic arch vessel configurations. Based on the compiled dimensions, these idealized models collectively are estimated to capture the major anatomic features found in the healthy adult aorta, represented by the two most common arch variants. Our choice of the healthy population has been inspired by the significance of aortic hemodynamics as addressed in Section 1. These CAD models are suitable for prudent use in hemodynamics and thrombi transport studies in both experimental and computational settings.

In this study, we performed an assessment of hemodynamics within the developed models in a left ventricular assist device (LVAD) setting using high-resolution CFD simulations and high-resolution planar PIV experiments. The flow pattern closely agrees between simulations and experiments at two flow conditions, as shown in the qualitative and quantitative comparisons. Additionally, we compared the flow within the idealized models with that of the four patients at an inflow condition of 5 LPM, which is the most common flow setting for the device and is also considered the cycle-averaged flow rate in a healthy cardiac cycle. The global flow features captured by the idealized models are generally representative of the patients. Notably, we did not present the results of experiments for patient-specific models due to the difficulties and challenges faced with optical imaging. Indeed, one motivation for developing idealized models has been the difficulties that encounter with optical imaging through patient-specific models, particularly in models fabricated using the layer-by-layer process of 3D-printing. The refractive index of each layer may differ slightly from the neighboring layers and the rest of the model, leading to local amplification of optical distortion by the arbitrary shape and surface quality in subject-specific models. An idealized model, with its simpler geometry and inherently better surface smoothness, minimizes such distortions.

This study has focused on developing idealized models that capture the general anatomical features of the aorta, rather than creating customized models tailored to specific pathological conditions or individual patient characteristics. However, the proposed models serve as the base geometry and can be customized to incorporate specific dimensional parameters for achieving various morphological geometries, including models for different diseases. These open-access idealized models can be easily modified by users to accommodate aortic diseases such as abdominal aortic aneurysm (AAA), ascending aortic aneurysm, and aortic coarctation.

The present study has certain limitations, most notably the absence of a comprehensive statistical analysis within the existing literature on morphometric parameters. Performing such an analysis would require a complex regularization of measurement resolution and addressing uncertainties associated with various imaging modalities and cadaveric studies falls outside the scope of our study. Furthermore, the idealized models were constructed using compiled dimensions from different studies, each focusing on a specific part of the aorta. In an alternative approach, upon the availability of a single large dataset of CT- or MRI-based scans of the full thoracic aorta, the authors envision image segmentation (manually or using AI-assisted methods) to extract all the detailed dimensions required to develop and formulate idealized models.

Additionally, for practical reasons, the flow assessment for all the cases was performed using steady-state flow conditions and rigid vessel walls. It is acknowledged that hemodynamics in the aorta is sensitive to inflow and outflow boundary conditions as well as vessel wall compliance. Depending on the purpose and metric under study, various parameters and study strategies become important at different levels. Generally, a pulsatile flow would cause a more complex flow pattern and affect flow unsteadiness and instabilities, vortex formation, transport, and breakdown. However, there is a complex interaction between different parameters, most notably the Reynolds number, Womersley number, and Strouhal number, e.g., see Peacock et al. (97). In terms of the wall compliance, rigid models commonly tend to generate increased systolic pressure, peak velocities, and pulse wave velocity, but depending on various parameters at play, the effects may be more or less pronounced, e.g., see Zimmermann et al. (15).

## 7 Conclusions

Our comprehensive study integrates literature-derived model design parameters to create two CAD models representing the aorta in healthy adults, specifically focusing on Normal and Bovine variants. The hemodynamics study within our idealized models under two left ventricular assist device (LVAD) support configurations showed that the global flow patterns in the idealized models agree well both qualitatively and quantitatively between CFD simulations and PIV experiments. Additionally, the flow structure is consistent between the idealized and the patient-specific cases in CFD simulations performed at a common LVAD support setting. This verification affirms the capability of our idealized models to replicate general flow features in subject-specific geometries. These meticulously constructed CAD models, rooted in statistical data, not only serve as valuable tools for investigating hemodynamics or solid mechanics but also hold promise for applications in medical device design where anatomical statistics are needed. The work contributes a robust foundation for advancing research in cardiovascular biomechanics and has implications for clinical and engineering domains.

## Data Availability

The CAD models can be found at the link provided in the Supplementary Material. The hemodynamics data is available upon request from the corresponding author.
