# Supplementary material for: Development of idealized human aortic models for in vitro and in silico hemodynamic studies
Source: Front Cardiovasc Med. 2024 Aug 5;11:1358601. doi: 10.3389/fcvm.2024.1358601 (PMC11330894; doi:10.3389/fcvm.2024.1358601)
Supplement: Supplementary file 3 [file Image1.pdf]

Supplementary Material

SUPPLEMENTARY FIGURES

A detailed statistical comparison between CFD simulations and PIV experiments for idealized models is presented in Figure S1. The left panel shows the probability density function (PDF) of the normalized velocity magnitude. The right panel illustrates the median values along with the 25% and 75% percentiles. It is important to note that, to ensure a fair comparison, the CFD data was extracted from a plane comparable to the PIV plane. While there are certain minor differences, the trends of the probability distributions and the velocity magnitude bounds remain consistent.

The PDFs and percentiles for velocity magnitude, vorticity magnitude, wall shear stress, and static pressure are presented in Figure S2. They showcase a detailed quantitative comparison between idealized models and patient-specific models for all CFD cases at the 5 LPM flow rate setting. The idealized models effectively capture the observed trends and bounds in the subject-specific models.

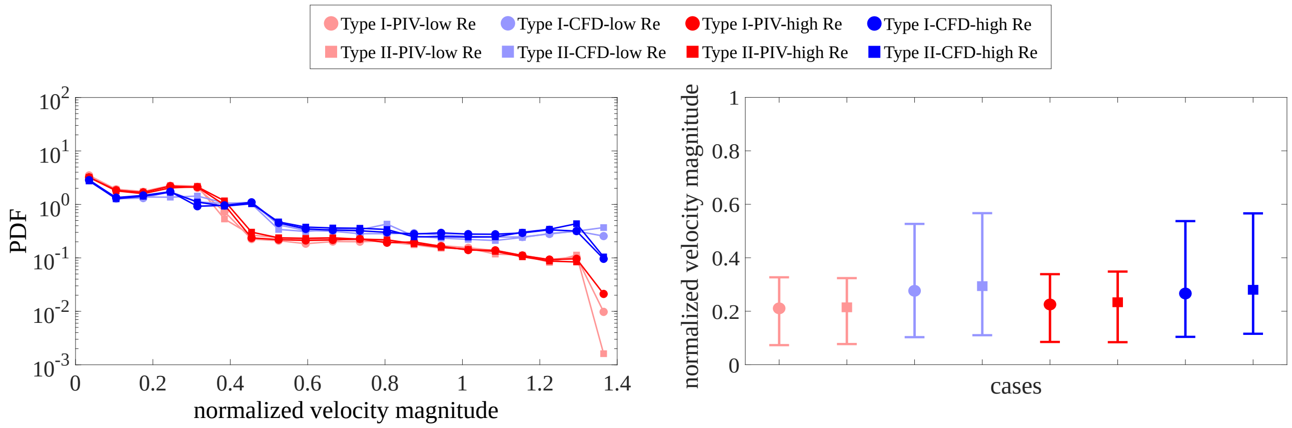

Figure S1. Left: probability density function (PDF) of the normalized velocity magnitude in idealized models obtained by CFD and PIV. Right: median of the normalized velocity magnitude along the 25% and 75% percentiles. Velocity magnitude is normalized by the inflow bulk velocity of each case. The Reynolds number (based on the inflow bulk velocity and the cannula diameter) is approximately 1757 and 3163 for the low and high flow rates cases, respectively.

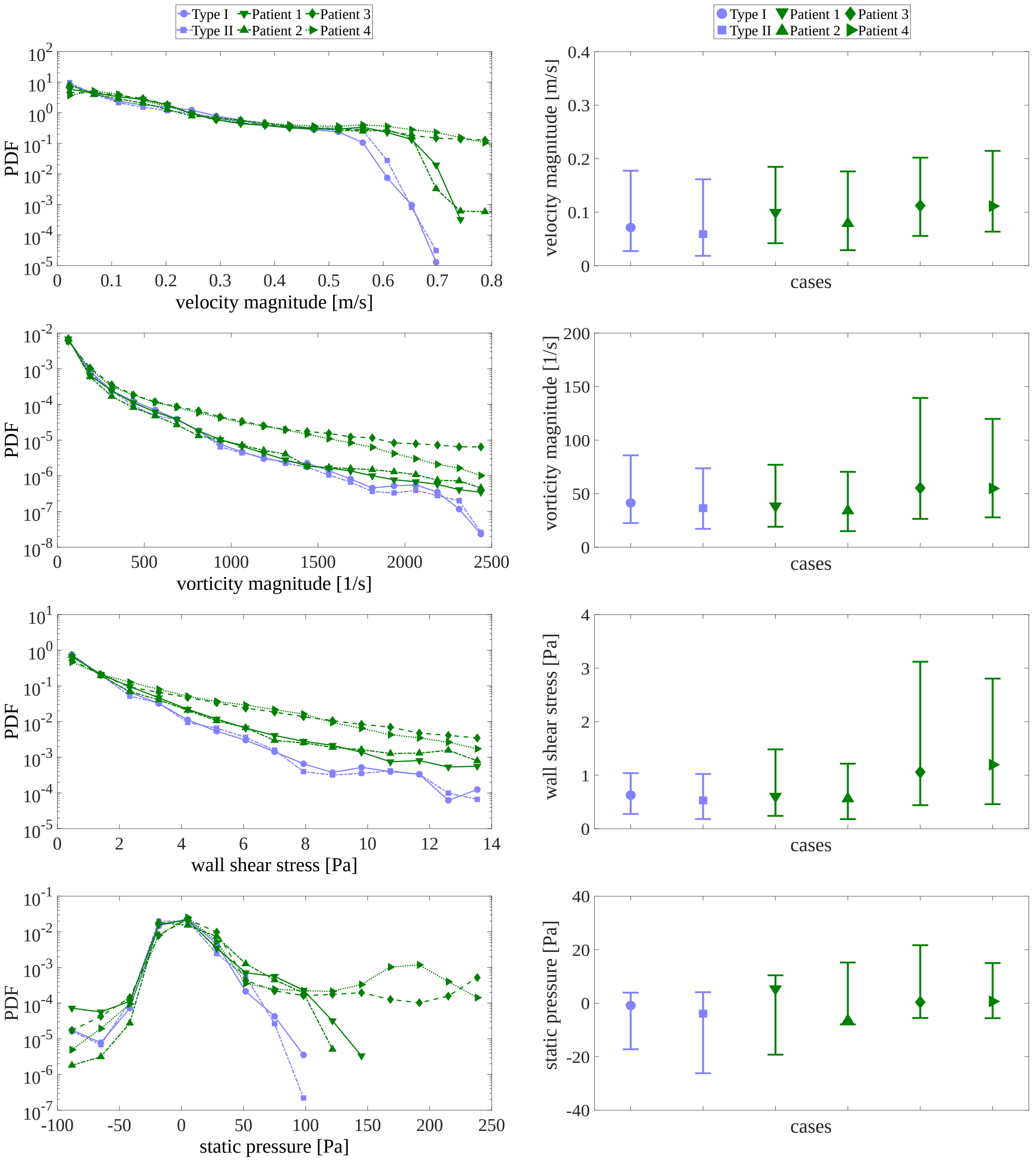

Figure S2. Plots of the probability density function (PDF) and percentiles (medians shown with the 25% and 75% percentile bounds) for velocity magnitude, vorticity magnitude, wall shear stress, and static pressure in both idealized models and patient-specific models obtained by CFD simulations. In all cases, the Reynolds number (based on the inflow bulk velocity and the cannula diameter) is approximately 1757.
